# Deep Learning-Based Automated Measurement of Murine Bone Length in Radiographs

**DOI:** 10.3390/bioengineering11070670

**Published:** 2024-07-01

**Authors:** Ruichen Rong, Kristin Denton, Kevin W. Jin, Peiran Quan, Zhuoyu Wen, Julia Kozlitina, Stephen Lyon, Aileen Wang, Carol A. Wise, Bruce Beutler, Donghan M. Yang, Qiwei Li, Jonathan J. Rios, Guanghua Xiao

**Affiliations:** 1 Quantitative Biomedical Research Center, Peter O’Donnell Jr. School of Public Health, The University of Texas Southwestern Medical Center, Dallas, TX 75390, USA; ruichen.rong@utsouthwestern.edu (R.R.); kevin.jin@utsouthwestern.edu (K.W.J.); peiran.quan@utsouthwestern.edu (P.Q.); zhuoyu.wen@utsouthwestern.edu (Z.W.); aileen.wang@utsouthwestern.edu (A.W.); donghan.yang@utsouthwestern.edu (D.M.Y.); 2 Center for Pediatric Bone Biology and Translational Research, Scottish Rite for Children, Dallas, TX 75219, USA; kristin.denton@utsouthwestern.edu (K.D.); carol.wise@utsouthwestern.edu (C.A.W.); 3 McDermott Center for Human Growth and Development, The University of Texas Southwestern Medical Center, Dallas, TX 75390, USA; julia.kozlitina@utsouthwestern.edu; 4 Center for the Genetics of Host Defense, The University of Texas Southwestern Medical Center, Dallas, TX 75390, USA; stephen.lyon@utsouthwestern.edu (S.L.); bruce.beutler@utsouthwestern.edu (B.B.); 5 Department of Orthopaedic Surgery, The University of Texas Southwestern Medical Center, Dallas, TX 75390, USA; 6 Department of Pediatrics, The University of Texas Southwestern Medical Center, Dallas, TX 75390, USA; 7 Department of Mathematical Sciences, The University of Texas at Dallas, Richardson, TX 75083, USA; qiwei.li@utdallas.edu; 8 Simmons Comprehensive Cancer Center, The University of Texas Southwestern Medical Center, Dallas, TX 75390, USA; 9 Department of Bioinformatics, The University of Texas Southwestern Medical Center, Dallas, TX 75390, USA

**Keywords:** keypoint detection, deep learning, mouse models

## Abstract

Genetic mouse models of skeletal abnormalities have demonstrated promise in the identification of phenotypes relevant to human skeletal diseases. Traditionally, phenotypes are assessed by manually examining radiographs, a tedious and potentially error-prone process. In response, this study developed a deep learning-based model that streamlines the measurement of murine bone lengths from radiographs in an accurate and reproducible manner. A bone detection and measurement pipeline utilizing the Keypoint R-CNN algorithm with an EfficientNet-B3 feature extraction backbone was developed to detect murine bone positions and measure their lengths. The pipeline was developed utilizing 94 X-ray images with expert annotations on the start and end position of each murine bone. The accuracy of our pipeline was evaluated on an independent dataset test with 592 images, and further validated on a previously published dataset of 21,300 mouse radiographs. The results showed that our model performed comparably to humans in measuring tibia and femur lengths (R^2^ > 0.92, *p*-value = 0) and significantly outperformed humans in measuring pelvic lengths in terms of precision and consistency. Furthermore, the model improved the precision and consistency of genetic association mapping results, identifying significant associations between genetic mutations and skeletal phenotypes with reduced variability. This study demonstrates the feasibility and efficiency of automated murine bone length measurement in the identification of mouse models of abnormal skeletal phenotypes.

## 1. Introduction

In vertebrates, the development of the skeleton is a highly regulated and intricate process. It relies on the coordinated activity of various genes and molecular mechanisms. This process is crucial for the organism’s survival and function because the skeletal system offers structural support, shields vital organs, and enables movement. Nevertheless, this intricate process can be disrupted by external environmental factors and internal genetic factors, resulting in the emergence of abnormal skeletal traits. Even seemingly small changes in the genome can propagate through downstream effects and result in abnormal phenotypes. In previous research, scientists have started to investigate how mouse models can help us understand the genetic factors influencing skeletal growth. Rios et al. established the feasibility of utilizing mice harboring N-ethyl-N-nitrosourea (ENU)-induced alleles to identify genetic determinants of skeletal growth, in which their analysis relied on manual examination of radiographs [1,2]. However, this is a time-consuming process requiring expert knowledge and can potentially lead to the introduction of human error [3]. 

In recent years, the rapid evolution of machine learning and medical imaging technologies has sparked a surge of interest in their application to medical image analysis. Computer vision algorithms comprise a subset of machine learning methods that leverage computational algorithms to automate the analysis and derive insight from visual stimuli. The advent of computer vision methods, especially ones based on deep learning, has enabled the automation and accurate analysis of medical images [4,5,6,7,8,9,10]. This automation not only significantly boosts analysis efficiency but also mitigates the risk of human errors. With the adoption of deep learning algorithms, healthcare practitioners can expedite disease diagnosis and treatment planning with heightened precision, enhancing the overall efficiency and accuracy of medical diagnostics.

Object detection is one of the core tasks in computer vision and finds extensive applications across various domains of medical image analysis. For example, in pathological image analysis, object detection can be used to automatically identify and label key features, such as tissue structures, cell nuclei, and organelles, aiding pathologists in rapid and accurate diagnosis and assessment of lesions [11,12]. Similarly, in radiology, object detection assists in automatic analysis by detecting and locating abnormal structures, lesions, and tumors in medical images like X-rays [13,14,15], CT scans [16,17,18], and MRIs [19,20,21]. Particularly relevant are the advances made in the field of AI-assisted radiology, which has demonstrated enormous clinical promise. Object detection algorithms applied to radiology can increase efficiency, reduce errors, and derive insight with minimal manual inputs [22]. 

Currently, object detection algorithms fall into two main categories: traditional machine learning methods and deep learning methods. Traditional methods, relying on manually designed features and classifiers, can limit performance. In contrast, deep learning methods, such as transformers and convolutional neural networks (CNNs), have revolutionized object detection. Examples include DETR [23,24,25], YOLO series [26,27,28,29,30], and R-CNN series [31,32,33,34]. DETR, based on a transformer architecture, transforms the object detection task into a sequence-to-sequence transformation. It employs a CNN encoder to convert input images into feature vectors and utilizes self-attention mechanisms in the decoder to capture global context, generating sequences of target categories and bounding boxes. YOLO series exemplify one-stage object detection algorithms, predicting object positions and categories directly from images without explicit region extraction steps; they are known for their simplicity and efficiency, often achieving faster detection speeds. On the other hand, the R-CNN series comprises typical two-stage object detection algorithms, first extracting candidate regions and then classifying and locating these regions, offering higher detection accuracy, albeit with increased computational complexity.

Of notable mention within the R-CNN series is Mask R-CNN. It operates in two main stages. In the first stage, it scans the image and employs the Region Proposal Network (RPN) to generate candidate regions, which serve to indicate potential object locations. In the second stage, each candidate region undergoes classification and bounding box regression, while simultaneously producing pixel-level masks for each object. By employing a multi-task learning approach, Mask R-CNN optimizes simultaneously for object detection, bounding box regression, and mask prediction. This method aids in enhancing the model’s performance by facilitating the sharing of convolutional features and reducing computational costs. Extending the Mask R-CNN framework, Keypoint R-CNN is tailored for keypoint detection tasks, such as human pose estimation and facial landmark detection. It accurately detects keypoints, offering detailed pose information crucial for various applications. The continual evolution of R-CNN algorithms has empowered medical image analysis, enhancing diagnostic accuracy and efficiency. We sought to apply this powerful model to automate the analysis of murine skeletal phenotypes; specifically, we developed a computer vision pipeline that automates the measurement of bone lengths in murine radiographs.

To overcome the tedium and inaccuracies of manual examinations in murine skeletal phenotype analysis, we have introduced a novel approach. Our contribution involves the development of a murine bone length measurement pipeline with Keypoint R-CNN [34]. This pipeline automates detecting mice, annotating keypoints, and measuring bone lengths for the radiographic images (X-rays). Our experiments indicate that the pipeline can provide fast and accurate measurement for bone length measurement (error < 0.05 pixels), and the phenotype analysis with pipeline results is consistent with manual measurements.

The remaining sections of the paper are summarized as follows: In Section 2.1, we introduce our dataset and the relevant preparatory work. We provide a detailed explanation of our proposed Bone Length Measurement Model in Section 2.2 and describe the training and implementation process in Section 2.3. In Section 3, we present and analyze our experimental results. Finally, we conclude with a discussion and conclusion in Section 4 and Section 5, respectively.

## 2. Materials and Methods

### 2.1. Datasets and Preparation

#### 2.1.1. X-ray Datasets and Annotations for Algorithm Design

**Figure 1:**
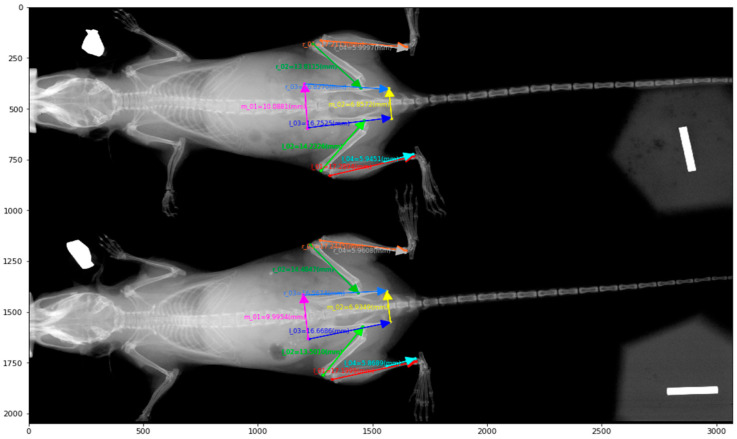
A Sample X-ray image and its annotations for bone length measurements.

We first scanned 94 radiographs from the dorsal view, with each image comprising either one or two mice. The start and end positions of each bone were manually labeled by experts; a sample annotation image can be found in Figure 1.

The dataset was split into 64 training images (derived from 126 mice), 11 validation images (from 22 mice), and 19 testing images (from 37 mice). The start and end coordinates of each bone were manually annotated with Hologic software (Marlborough, MA, USA) and used as ground truth keypoints. We then prepared an independent testing dataset with 592 radiographs with manually labeled start and end positions of each bone (Table 1).

#### 2.1.2. Mouse Mutagenesis, Genotyping, and Radiography 

The mouse mutagenesis and breeding process was performed under the protocol in Wang’s publication [35]. All procedures were approved by the Institutional Animal Care and Use Committee at the University of Texas Southwestern Medical Center.

Male mice were treated with the mutagen ENU, then out-crossed to non-mutagenized C57BL/6J female mice. Nonsynonymous ENU-induced alleles were detected by exome sequencing the resulting male pups (Generation G1). These G1 founder males were out-crossed to C57BL/6J females, and resulting G2 females were back-crossed to their G1 male founder. All resulting G3 mice were genotyped by massively parallel targeted sequencing. 

Automated meiotic mapping (i.e., linkage mapping) was performed within the Mutagenetix database. Statistically significant associations between ENU alleles and residual variation in skeletal phenotypes were identified following Bonferroni correction for the number of ENU alleles analyzed in the pedigree [1]. Associated loci were further evaluated using the *Candidate Explorer* tool [36], a machine learning algorithm that integrates dozens of in silico, genetic, and biologic features to implicate the causality of individual loci for the associated phenotype. 

For radiographic imaging, mice were anesthetized by inhaled isoflurane, and X-ray radiography was performed using an UltraFocusDXA instrument (Hologic Inc., Marlborough, MA) with continued anesthesia [1,2]. Radiographic parameters (i.e., bone lengths) were measured manually with Hologic software. For each mouse, we measured the tibia, femur, and pelvic length on both the left and right sides. Radiographically measured bone lengths were compared to expected bone lengths using statistical models adjusting for age and gender, which were developed using the radiographs of 25,300 mice [1]. Residual differences between the measured and expected bone lengths were used for automated meiotic mapping.

### 2.2. Bone Length Measurement Model

#### 2.2.1. Keypoint Detection 

**Figure 2:**
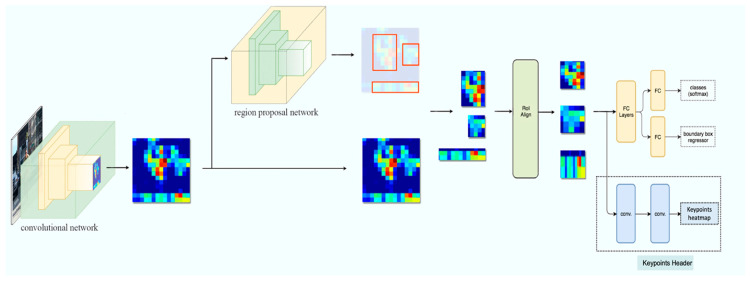
Overview of the Keypoint R-CNN bone measurement pipeline.

We built our bone length measurement model with object detection and keypoint detection algorithms. We utilized the Keypoint R-CNN algorithm to automatically detect the mice, their position, and the start and end coordinates of each bone in each mouse. Keypoint R-CNN is a two-stage object detection algorithm consisting of a feature extraction backbone, an objectness ROI header, and a keypoint detection header. Our model architecture is depicted in Figure 2.

Feature extraction backbone architecture: In our approach, we used the EfficientNet-B3 [37] model as the feature extractor on top of the framework of the Feature Pyramid Network (FPN) [38]. The EfficientNet-B3 network, a member of the EfficientNet family, optimizes the model in various aspects by effectively utilizing scaling factors of network depth, width, and resolution. While maintaining a concise model structure, EfficientNet-B3 fully utilizes limited parameters to express richer feature information, which makes it maintain a good balance between high accuracy and smaller model size, leading to higher efficiency. Compared to widely used models like ResNet [39] and VGG [40], EfficientNet-B3 requires fewer parameters and computational resources, making it an ideal choice for handling large-scale data and operating in resource-constrained environments. FPN employs an efficient multi-scale feature fusion approach as its core principle, integrating high-level semantic information with low-level detailed information through operations such as bottom–up, top–down, and lateral connections. This strategy effectively enhances the scale robustness of feature representation, resulting in richer and more comprehensive feature maps across scales. FPN can adapt to target detection tasks of varying scales and morphologies while achieving significant performance improvements without substantially increasing computational overhead. This combined structure not only improves computational efficiency but also further enhances the detection accuracy of keypoint detection.

- Objectness ROI header: The objectness ROI header attempts to detect the mouse’s position and extract the region-of-interest (ROI) containing the target keypoints. We utilized the default MultiAlign-ROI implementation from the Faster R-CNN algorithm [25]. The MultiAlign-ROI technique maps candidate regions of varying sizes, generated by the region proposal network, to a fixed-size feature map. In contrast to traditional methods, MultiAlign-ROI utilizes bilinear interpolation, a method of interpolation that estimates new pixel values by taking a weighted average of surrounding pixel values in an image, for precise alignment of ROIs to the feature map grid, thereby mitigating spatial misalignment and enhancing the accuracy of object detection and recognition.
- Keypoint detection header: The keypoint detection header locates the keypoint coordinates inside the ROI produced by the objectness ROI header. It transfers the backbone feature maps within the ROI region into heatmaps and returns the coordinates with the brightest points. The network was constructed with 8 repeats of convolution + BatchNormalization + Activation blocs, followed by a transposed convolution layer to generate the keypoint coordinates and confidence scores.
- Loss functions: We utilized a combined loss function of objectness loss, class loss, and keypoint loss.
- Objectness loss is a cross-entropy loss used to measure the accuracy between mouse objects and non-mouse objects. Specifically, objectness loss quantifies the model’s performance by computing the difference between the predicted probability distribution of mouse objects and non-mouse objects and the actual labels. For each predicted bounding box, the model outputs a probability distribution indicating whether the box contains a mouse object. Objectness loss compares the model’s predictions with the ground truth labels, assigning lower loss if the prediction is correct and higher loss otherwise.
  (1) $$L_{Objectness}=-\frac{1}{N}\sum_{i=1}^{N}\left[y_{i}\log\left(p_{i}\right)+\left(1-y_{i}\right)\log\left(1-p_{i}\right)\right]$$
  where *N* is the total number of predicted bounding boxes. $y_{i}$ is the ground truth label for the ith predicted bounding box. It equals 1 if the box contains a mouse object (positive sample) and 0 otherwise (negative sample). $p_{i}$ is the predicted objectness score for the ith bounding box, representing the confidence that the box contains a mouse object.

- Class loss evaluates the accuracy of classification by comparing the predicted class and the actual class labels. For each detected object, the model generates a probability distribution indicating the likelihood of belonging to each class. Class loss compares the model’s predicted probability distribution with the one-hot encoding of the actual class labels and calculates their cross-entropy to assess classification accuracy. In this paper, we use cross-entropy loss to measure the accuracy of the model’s predicted mouse body positions. By minimizing class loss, the model can learn more discriminative features to distinguish between different object categories. This helps improve the accuracy and robustness of the object detection system, enabling it to accurately identify and classify objects.
  (2) $$L_{class}=-\frac{1}{N}\sum_{i=1}^{N}\sum_{c=1}^{C}y_{i,c}\log\left(p_{i,c}\right)$$
  where *N* is the total number of predicted bounding boxes. C is the total number of classes. $y_{i,c}$ is the ground truth label for the ith predicted bounding box and cth class. It equals 1 if the box belongs to class c and 0 otherwise. $p_{i,c}$ is the predicted probability that the ith bounding box belongs to class c.

- Keypoint loss evaluates the accuracy of predicted keypoint coordinates by comparing them with the actual labeled keypoint coordinates. For each keypoint, the model generates a predicted coordinate representing its position. Keypoint loss assesses the prediction accuracy by calculating the mean squared error (MSE) between the predicted and actual coordinates. MSE reflects the distance between the predicted and actual values, indicating the accuracy of the prediction. By minimizing keypoint loss, the model can learn to predict object keypoints more accurately. This helps improve the model’s performance in tasks such as pose estimation and keypoint detection, enabling it to accurately locate and identify key parts of objects.
  (3) $$L_{keypoints}=-\frac{1}{N}\sum_{i=1}^{N}\sum_{k=1}^{K}{\left\|{pred}_{i,k}-{gt}_{i,k}\right\|}_{2}^{2}$$
  where *N* is the total number of predicted bounding boxes. K is the total number of keypoints per bounding box. ${pred}_{i,k}$ is the predicted coordinates of the kth keypoint for the ith bounding box. ${gt}_{i,k}$ is the ground truth coordinates of the kth keypoint for the ith bounding box.

Our loss is the summation of the objectness loss, class loss, and keypoint loss.

(4) $$L=L_{Objectness}+L_{class}+L_{keypoints}$$

#### 2.2.2. Pre-Processing

We pre-process the images and annotations into a format suitable for model input. To mitigate overfitting and enhance the performance of the model, we employ a series of data augmentation techniques, including shift-scale rotation, random horizontal flipping, random vertical flipping, and random adjustment of brightness and contrast. This diverse set of augmentations ensures that the model is exposed to a wide variety of variations in the data, promoting robustness and generalization in the trained model.

#### 2.2.3. Post-Processing

The model outputs the following information for each image: (1) Location of a detected mouse; (2) The probability of the image being a top-view; (3) The coordinates of keypoints in each detected object. We only retained objects with >0.5 probability of being a top-view mouse and extracted the coordinates of their key points. We then assigned key points to the beginning and end positions of each bone and measured its length. We further refined the detection results by removing outliers and identifying abnormal bone shapes based on their length and relative location. 

### 2.3. Implementation

We implemented Keypoint R-CNN using the official torchvision [41] object detection framework and the Adam optimizer to update model parameters. We began with a warm start learning rate of 0.0002 for one epoch and progressed to an initial learning rate of 0.001 with multi-step learning rate decay for the rest of the training. Since the datasets for training mice position and training keypoints have different ground truths, feeding both datasets together into the model would lead to a loss value of NaN. During training, we alternated between each dataset and updated their corresponding headers. We did so by first freezing all parameters in the keypoint detection header and only using position detection data to calculate objectness loss with class loss to update the backbone and ROI header parameters. Then, we froze all parameters in the ROI header and only used keypoint detection data to calculate objectness loss and keypoint loss to update the backbone and keypoint header parameters. We trained each header until the position accuracy and keypoint MSE on the validation dataset plateaued. The object detection header was trained to 100 epochs, and the keypoint header was trained to 1000 epochs. We ran our experiment on an NVIDIA tesla V100 GPU node (with 32 GB of memory) with a batch size of 4. 

### 2.4. Evaluations

We calculated the average pixel-level MSE between the ground truth keypoints and predicted keypoints to evaluate the keypoint detection accuracy. We conducted correlation analyses for both the ground truth bone length and the algorithm-calculated bone length to evaluate the measurement performance. 

## 3. Results

### 3.1. Keypoint Detection Accuracy

We evaluated the keypoint detection accuracy by calculating the mean squared error (MSE) between ground truth coordinates and model-predicted coordinates for annotated keypoints (Table 2). Our model correctly detected the number of distinct mice in each image. The overall MSE distance between the ground truth and prediction was consistently within 0.05 pixels for both validation and testing datasets, indicating high precision in keypoint localization.

### 3.2. Bone Length Accuracy

**Figure 3:**
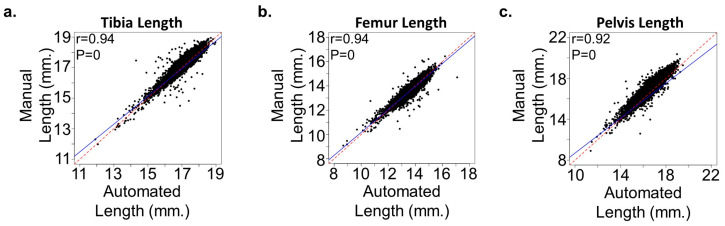
Correlation in bone lengths between the automated and manual approaches. (**a**–**c**) Plots demonstrate significant correlation between the automated and manual methods in measuring the (**a**) tibia, (**b**) femur, and (**c**) pelvic length in mice (n = 21,300). Regression lines (solid blue) are shown compared to a perfect correlation (red dash). The Pearson correlation coefficient (r) is shown in each plot.

We tested our algorithm on a large testing dataset with 1178 mice in 592 X-ray images. This dataset does not contain the keypoint information for each bone but does contain the bone lengths manually measured with Hologic software. We calculated the bone length based on the keypoint detection result and post-processing step. Figure 3 shows the correlation analysis between machine-calculated bone lengths and manually measured bone lengths, demonstrating high consistency (R^2^ > 0.92, *p*-value = 0) across different bones. This high correlation indicates that the automated measurements are highly reliable, providing a robust alternative to manual measurements, which can be both time-consuming and prone to human error.

### 3.3. Consistency across a Large Discovery Cohort

**Figure 4:**
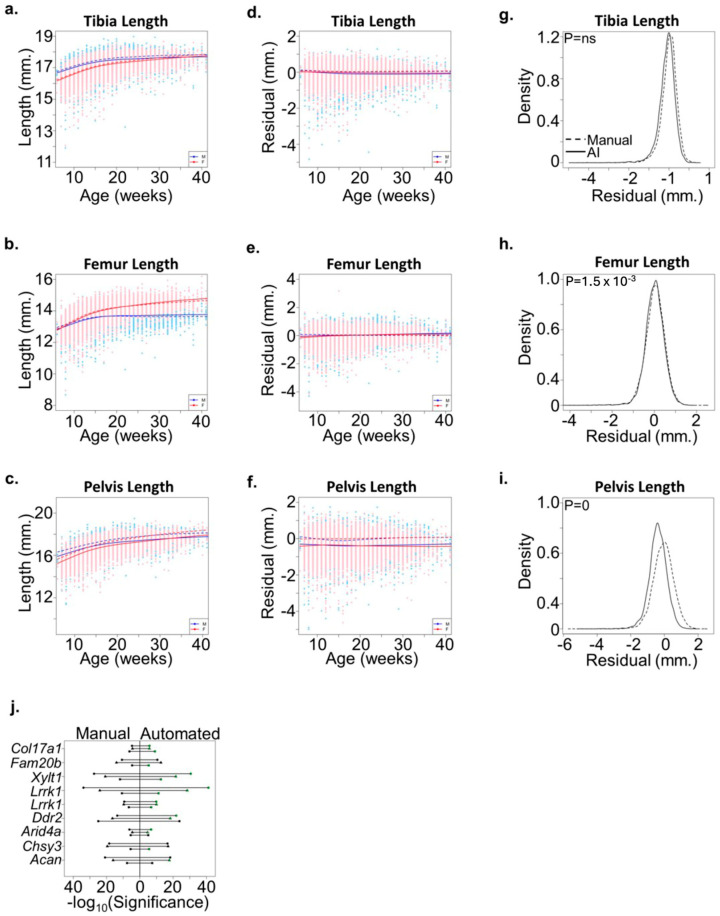
Validation of the automated method. (**a**–**c**) Plots demonstrate the phenotypic relationship between age and gender for (**a**) tibia length, (**b**) femur length, and (**c**) pelvic length among 21,300 mice analyzed by the automated method. Smoothing lines show that the automated measurements (solid lines) are similar to previously published manual measurements (dashed line). Male (M) and female (F) are shown separately. (**d**–**f**) Plot of (**d**) tibia, (**e**) femur, and (**f**) pelvic length residuals following adjustment for age and gender. Smoothing lines demonstrate that the automated method (solid line) results are similar to the manual method (dashed line) for tibia and femur lengths but are systematically reduced for pelvic length. Male (M) and female (F) are shown separately. (**g**–**i**) Density plots of (**g**) tibia, (**h**) femur, and (**i**) pelvic length residuals using the automated (solid line) and manual (dashed line) methods. Statistically significant differences in the variances between both methods were evaluated using the F-test. (**j**) Plot showing statistical significance following linkage analysis of nine alleles in eight genes associated with variations in the tibia (circle), femur (triangle), or pelvic (square) length using either the manual (left) or automated (right) method. Phenotypes with improved statistical significance using the automated method are shown in green.

To further evaluate the accuracy of bone measurement predicted by the automated method, we re-analyzed radiographs from 21,300 mice in a study previously published [1]. The high correlation between manual and automated methods (Figure 3) was consistent across male and female mice of different ages (Figure 4a–f). Interestingly, automated measures of pelvic length were consistently less than manual measures in this cohort (Figure 4c,f). This discrepancy highlights the increased precision of the automated method in consistently detecting bone ends, which can be challenging in manual measurements due to variability in human perception. Accurate measurement of the pelvic length requires precise and reproducible identification of the bone ends. We hypothesized the machine learning approach would more consistently detect bone ends and reduce the overall variability compared to the manual measurement approach. To test this hypothesis, we compared the variances between the manual and automated approaches for all skeletal measures. No significant difference was detected for the tibia length measurement, and only a slight but significant difference was observed for femur length (Figure 4g,h). In contrast, the automated approach significantly reduced the variance in pelvic length residual measures compared to the manual approach (Figure 4i). These results highlight the added precision for which the automated method reliably segments skeletal features and reduces variability introduced by manual measurements. 

Additionally, to assess whether the automated method altered genetic association mapping to ENU alleles, we compared automated meiotic mapping results for selected alleles with “good” or “excellent” candidate annotations using the Candidate Explorer tool [29]. We identified nine alleles associated with phenovariance for at least two skeletal phenotypes including pelvic length, located in genes previously implicated in skeletal development and for which results could be directly compared between methods. For most alleles, association results improved for at least two skeletal phenotypes (Figure 4j). Taken together, these results suggest that the precision and reproducibility of the automated method improve the detection of ENU alleles associated with variations in skeletal development. These improvements indicate that the automated method not only matches but can surpass manual methods in certain contexts, providing more reliable data for genetic studies and potentially uncovering new insights into skeletal development.

## 4. Discussion

In this study, we developed a deep learning-based model to automate the measurement of murine bone lengths from radiographs. The results demonstrate the effectiveness of our automated pipeline by achieving high accuracy and reproducibility in detecting murine bone positions and measuring their lengths. This automation significantly reduces the time and effort required for manual examination and measurement, making large-scale studies feasible. The model performed on par with human experts in measuring tibia and femur lengths, while outperforming humans in precision and consistency when measuring pelvic lengths. These findings indicate the potential application of our automated approach in improving the accuracy and reliability of skeletal phenotype measurements.

Furthermore, the streamlined process minimizes potential errors introduced by human observers and could prove useful in the discovery of novel genetic mouse models with abnormal skeletal phenotypes. The improved precision and consistency achieved by our model has practical implications in genetic association mapping and has demonstrated its reliability in genetic association studies regarding murine bone length variations associated with abnormal skeletal phenotypes. This suggests that our automated pipeline can contribute to more robust and accurate identification of genetic mouse models and to furthering our understanding of human skeletal diseases. Future research could expand our work by incorporating additional skeletal measurements and exploring the application of our automated pipeline to other animal models or clinical settings.

## 5. Conclusions

In conclusion, our novel deep learning-based approach for automating murine bone length measurements has shown promising results. The pipeline, utilizing Keypoint R-CNN, demonstrated fast and accurate measurements with minimal error. By streamlining the process, we significantly reduce manual effort, enabling large-scale studies and improving accuracy compared to manual examinations. The model’s performance, on par with human experts and surpassing them in precision and consistency for certain measurements, suggests its potential for enhancing skeletal phenotype analysis. Such improvements are crucial for genetic association mapping, where accurate phenotype measurements are essential. The practical implications of our findings extend to the discovery of novel genetic mouse models with abnormal skeletal phenotypes, potentially accelerating research into skeletal diseases. Additionally, the automated approach facilitates high-throughput screening in preclinical studies, contributing to the development of treatments for skeletal disorders. The efficiency and scalability of our pipeline make it suitable for extensive studies, providing deeper insights into genetic and environmental influences on skeletal phenotypes. Integrating this approach with other imaging modalities, such as MRI or CT scans, could further enhance the comprehensiveness and applicability of skeletal phenotype assessments in various research and clinical environments. Our study underscores the potential of deep learning-based automation in transforming skeletal phenotype analysis, offering a reliable and scalable solution that aligns with the demands of modern genetic research and clinical diagnostics.

## Figures and Tables

**Table 1:** The number of images and mice in each dataset. Most X-ray images comprise two mice.

	No. Images	No. Mice
Training	64	126
Validation	11	22
Testing	19	37
External Testing	592	1178

**Table 2:** The Keypoint detection performance of Keypoint R-CNN. The model correctly detects distinct mice in X-rays and limits the keypoints location error within 0.05 pixels.

	Objectness Accuracy	Keypoints MSE
Validation	1.0000	0.0257
Testing	1.0000	0.0242

## Data Availability

The original data presented in the study are openly available online at https://mutagenetix.utsouthwestern.edu.
